# Fast-moving dislocations trigger flash weakening in carbonate-bearing faults during earthquakes

**DOI:** 10.1038/srep16112

**Published:** 2015-11-10

**Authors:** Elena Spagnuolo, Oliver Plümper, Marie Violay, Andrea Cavallo, Giulio Di Toro

**Affiliations:** 1 Sezione di Sismologia e Tettonofisica, Istituto Nazionale di Geofisica e Vulcanologia, Via di Vigna Murata 605, Roma, Italy; 2 Department of Earth Sciences, Utrecht University, Budapestlaan, 4 P.O. Box 80.021, 3584 CD Utrecht, the Netherlands; 3 EPFL, LEMR, Station 18, 1015, Lausanne, Switzerland; 4 Dipartimento di Geoscienze, Università di Padova, Via G. Gradenigo 6, 35131 Padova, Italy; 5 School of Earth, Atmospheric and Environmental Sciences, Manchester University, Oxford Street, M13 9PL Manchester, United Kingdom

## Abstract

Rupture fronts can cause fault displacement, reaching speeds up to several ms^−1^ within a few milliseconds, at any distance away from the earthquake nucleation area. In the case of silicate-bearing rocks the abrupt slip acceleration results in melting at asperity contacts causing a large reduction in fault frictional strength (i.e., flash weakening). Flash weakening is also observed in experiments performed in carbonate-bearing rocks but evidence for melting is lacking. To unravel the micro-physical mechanisms associated with flash weakening in carbonates, experiments were conducted on pre-cut Carrara marble cylinders using a rotary shear apparatus at conditions relevant to earthquakes propagation. In the first 5 mm of slip the shear stress was reduced up to 30% and CO_2_ was released. Focused ion beam, scanning and transmission electron microscopy investigations of the slipping zones reveal the presence of calcite nanograins and amorphous carbon. We interpret the CO_2_ release, the formation of nanograins and amorphous carbon to be the result of a shock-like stress release associated with the migration of fast-moving dislocations. Amorphous carbon, given its low friction coefficient, is responsible for flash weakening and promotes the propagation of the seismic rupture in carbonate-bearing fault patches.

During an earthquake, at any distance away from the nucleation area, the passage of the rupture front results in fault slip reaching speeds up to several ms^−1^ within a few milliseconds^1^. The seismic rupture propagates because the fault strength decreases with increasing slip and slip rate upon the activation of a weakening mechanism. So far, frictional heating and thermal pressurization have been suggested as possible weakening mechanisms occurring at the initiation of slip in the case of both natural and experimental faults^2^,^3^. In natural silicate-bearing rocks pseudotachylites (solidified friction-induced melts) record frictional heating^4^ occurred during seismic slip events. Theoretical and experimental investigations^5^,^6^,^7^,^8^ have suggested that such frictional heat initiates at a few highly stressed microscopic asperities (flash heating), where abrupt temperature increase results in instantaneous melting (flash melting) at the asperity contact. The microscopic melt droplets produced at the initial stage of slip lubricate the fault and cause a reduction in frictional strength^9^. With progressive slip, the entire fault surface is covered by a continuous melt layer resulting in frictional melt lubrication^4^,^10^,^11^,^12^.

Faults cutting carbonate-bearing rocks produce moderate to large earthquakes (e.g., L’Aquila 2009 Mw 6.1 ref. 13, Wenchuan 2008 Mw 7.9 ref. 14). Moreover experiments performed on carbonates-bearing rocks approaching seismic deformation conditions^15^,^16^,^17^,^18^,^19^ report a 95% decrease of the initial experimental fault strength. Despite these evidences for fault weakening in the case of carbonate-bearing rocks, melt-related products were not observed and other processes were proposed to explain seismic rupture propagation.

Evidence for thermal decomposition^15^,^16^,^17^ (i.e., decarbonation vesicles) suggests that frictional heating occurs to activate thermochemical pressurization of fluids (CO_2_) leading to frictional strength reduction^15^,^16^. With progressive slip the transition to frictional weakening can be explained by nanoparticle and powder lubrication^18^,^19^,^20^,^21^, grain boundary sliding, nanometric flow and phase transformations^22^,^23^,^24^ or dynamic recrystallization^25^,^26^,^27^. All these proposed mechanisms rely on the presence of nanograins, crystal-plastic microstructures and mirror-like surfaces observed both in experimental and natural faults^26^,^27^,^28^. However, none of the aforementioned mechanisms are able to explain the processes operating at the micro-scale that produce the initial conditions for frictional heating, grain-size reduction and phase transformations, which are necessary to trigger fault weakening. In order to study the processes operating at the initiation of slip we sheared pre-cut samples of a 99% calcite Carrara marble to simulate the behaviour of a fault at conditions relevant for earthquake propagation. The experiments replicate the effect of an abrupt acceleration to seismic slip rates, which occurs when a part of the fault is subjected to a strong modification of the stress field (due to the rupture propagating from neighbouring points). The large surface frictional power density (1–100  MWm^−2^) dissipated into a low thermal diffusivity (~10^×6^ m^2^s^−1^) and a narrow (<cm) slipping zone resulted in intense rock fragmentation and in an abrupt temperature increase.

Dislocations as well as microcracks and grain boundaries have been previously studied in association with seismic activity during earthquakes^29^,^30^ and are responsible of reduced rock strength well below the theoretical strength of crystalline materials^31^. Dislocations moving at strain rates as fast as earthquake deformation rates may have an unpredictable effect on the frictional strength reduction as depicted by theoretical models for shock impacts and high speed friction^32^. When dislocations move at high speed the thermal softening associated with plastic deformation is hindered and brittle failure may occur^33^. Here we suggest that fast-moving dislocations within carbonate-bearing rocks cause the abrupt temperature increase, rock nano-fragmentation and the formations of patches of amorphous carbon that, in analogy with flash melting in the case of silicate-bearing rocks, trigger fault weakening at the initiation of seismic slip.

## Methods

Ten experiments (Table 1) were performed with SHIVA^34^ (Slow to High Velocity Apparatus, installed at INGV Rome) on 50/30 mm outer/inner diameter Carrara marble cylinders (grain size ~300 μm, >99% calcite, without amorphous carbon or graphite: for description of sample preparation see Nielsen *et al.*^35^). Mechanical data (axial load, torque, axial displacement, angular rotation) were acquired at a frequency of 25 kHz and given in terms of equivalent slip *δ*, slip-rate *V* and shear stress *τ* determined on the annular sample^36^. Experiments consisted of the following steps: (1) application of normal stress *σ*_*n*_ = 10 MPa, (2) slip acceleration of 8.5 ms^−2^ to the target velocity *V*_*tar*_ and (3) slip deceleration to halt the sample at a specific slip distance, with the exception of experiments s257 and s301, where the final slip was manually attained. Experiment s915-0 was loaded to 10 MPa but not sheared to investigate the effects of sample grinding and polishing plus the application of normal stress on the sliding surface. Samples were recovered after the experiment, coated with a Cr-nanolayer and examined within a field-emission scanning electron microscope (FE-SEM; JEOL JSM-6500F, INGV, Rome) equipped with an energy-dispersive X-ray spectrometer(EDS). Selected areas of the samples s915-0, s915-1 and s761 (arrested at slip of 0, 1.5 mm and 5 mm, respectively) were coated with a Pt-nanolayer and analysed in a focused ion beam SEM (FIB-SEM, FEI Nova 600 Nano Lab, Utrecht University). FIB-SEM nano-manipulations were carried out either to obtain (1) cross-sections parallel and perpendicular to the shear direction or (2) ultra-thin, electron-transparent foils for transmission electron microscopy (TEM, FEI Tecnai 20F and FEI Tecnai 12, Utrecht University) investigations (Supplementary Fig. S1 online). The scanning mode of the Tecnai 20F TEM was used to generate high-angle annular dark-field scanning TEM (HAADF- STEM) images. Gas emissions were measured with a Pfeiffer Quadstarquadrupole mass spectrometer. To detect gas (e.g.,CO_2_) variations with respect to the atmospheric content and to avoid gas dispersions, we performed a specific experiment (s1187), in which we imposed a succession of slip pulses (three of 1.5 mm and one of 50 mm of slip, respectively) on the rock sample inserted in a vessel^17^. In experiment s1187, between each slip pulse, we opened the vessel to polish the surfaces and restore atmospheric conditions.

## Results

**Figure 1:**
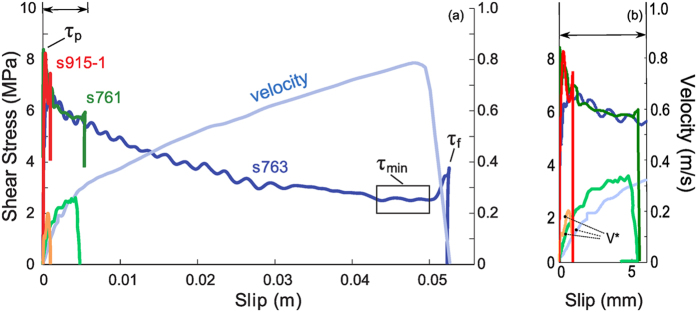
Mechanical data. Panel (**a**) Evolution of the shear stress and slip rate in experiments with increasing slips of 1.5 mm (red in colour), 5 mm (green), 50 mm (blue). Slip zones of experiments s915_1 (1.5 mm) and s761 (5 mm) were recovered for microstructural analysis (Figs 3, 4, 5). Panel (**b**) Zoom of the first 5 mm of slip where the velocity threshold for weakening (V*) is indicated by dashed lines.

**Figure 2:**
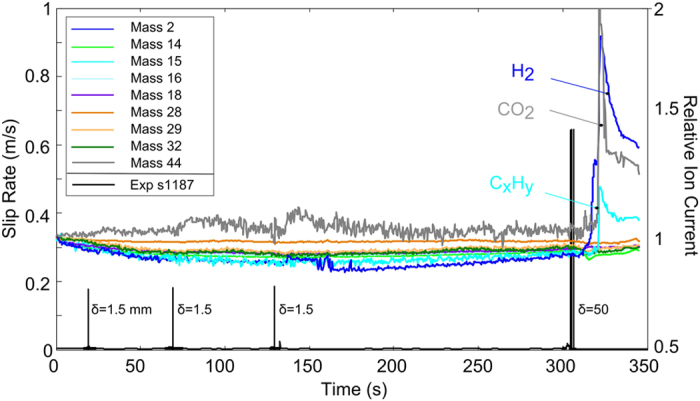
Emission spectra for selected masses recorded with a Pfeiffer Quadstarquadrupole mass spectrometer during three slip pulses of 1.5 mm and one of 50 mm (experiment s1187). Each slip pulse was performed at σ_n_ = 10 MPa, *V*_tar_ = 1 m/s and inside a vessel to capture gas emission from the shear zone. The diagram reports the ion current relative to the initial atmospheric background value. Significant variations are recorded in case of mass 2 (H_2_), mass 44 (CO_2_), mass 15 (C_x_H_y_). After each pulse the vessel was opened to remove the gouge from the sliding surfaces and to restore the initial atmospheric conditions. A delay was observed between the slip pulse and the detection of the compounds due to the travel time of the gas through the capillary and the acquisition time of the mass spectrometer.

We measured the shear stress (*τ*) at constant normal stress (*σ*_*n*_) which approximates the frictional fault strength during slip (Fig. 1). In all experiments *τ* first increased almost linearly to a peak value *τ*_*p*_ = 7.17 ± 1.0 MPa during the acceleration stage. A velocity threshold (V* = 0.12 ± 0.05 ms^−1^) for weakening was observed in all experiments after which shear stress reduction initiated in combination with macroscopic slip. A minimum shear stress value achieved at macroscopic slip was *τ*_min_ ~ 0.51 MPa after a total slip of 20 m. During the deceleration stage, the shear stress recovered from *τ*_*min*_to a final value *τ*_*f*_ whose magnitude increased with slip. CO_2_ concentration increased by 4% after 1.5 mm, 33% after 5 mm and 130% after 50 mm of slip compared to the background CO_2_ value of ~350 ppm. In experiment s1187, performed by shearing the rock samples inside an insulating vessel, the concentration of H_2_ (mass 2) and CO_2_ (mass 44) relative to the atmospheric level increased after each individual 1.5 mm slip pulse. A significant increase in C_x_H_y_ fragments (mass 15) was detected after the final 50 mm slip pulse (Fig. 2, given in terms of ion current). No significant variations in O_2_ (mass 32) were observed during the experiment. Calibration tests performed inside the vessel by replacing the rock samples with stainless steel cylinders confirmed that the detected gases resulted from the shearing of the Carrara marble. SEM imaging of the pre-sheared samples and sample surface sheared to 1.5 mm and 5 mm reveals a number of flattened micro-patches increasing in continuity and size with increasing slip (Supplementary Fig. S2 online). For *δ*_tot_ > 1.5 mm the sliding surface is smooth in agreement with observations from previously investigated carbonate sliding surfaces^25^,^26^,^27^.

**Figure 3:**
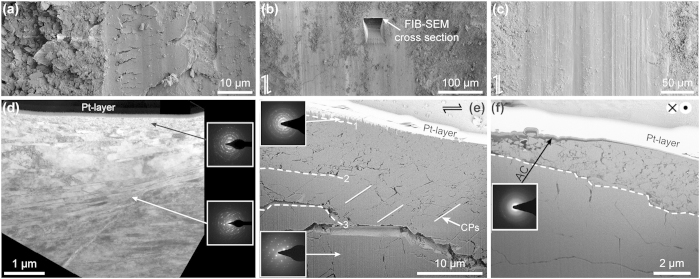
Evolution of the marble microstructures with increasing slip. Sliding surface after (**a**) 0 mm, (**b**) 1.5 mm, and (**c**) 5 mm slip (SEM images). (**a**) Rare incoherent flat micro-patches induced through diamond tool rock polishing in pre-sheared surfaces. (**d**) TEM image of a FIB-SEM section across the micro-patch in (**a**) reveals a nanograin volume (grain size >50 nm) covering bands of interleaving, elongated calcite grains. The remaining sample surface is free of nanograins (Supplementary Fig. S3 online). With increasing displacement flat slip surfaces (**b** and **c**) spread coherently across the sample. (**e**) A nanograin volume (Domain 1) develops below the slip surface covering extensively cleaved (CP: cleavage plane) and fractured calcite grains (Domain 2). The latter terminates into un-deformed calcite grains (Domain 3). Domains 1, 2, 3 are separated by dashed lines. (**f**) After 5 mm displacement the nanograin volume is covered by a semi-coherent amorphous carbon (**a**–**C**) layer.

The micro-patches of the pre-sheared rock surfaces (s915-0, *δ*_tot_ = 0.0 mm, Fig. 3a, Supplementary Fig. S3 online) present a flat-faced texture characterized by exposed cleavages, exfoliated fragments and extensive cracks parallel to the surface. TEM investigations of cross-sections excavated perpendicular to the flattened micro-patch reveal a 200 nm thick calcite nanograin layer (grain size: 40–100 nm) covering a deformation zone characterized by bands of interleaving, elongated calcite grains (Fig. 3d). However, the pre-sheared rock surface is dominated by the exposure of nanograin-free, calcite crystal surfaces implying that the incoherent grain micro-patches result from diamond tool rock polishing and application of normal stress (Supplementary Fig. S3 online).

**Figure 4:**
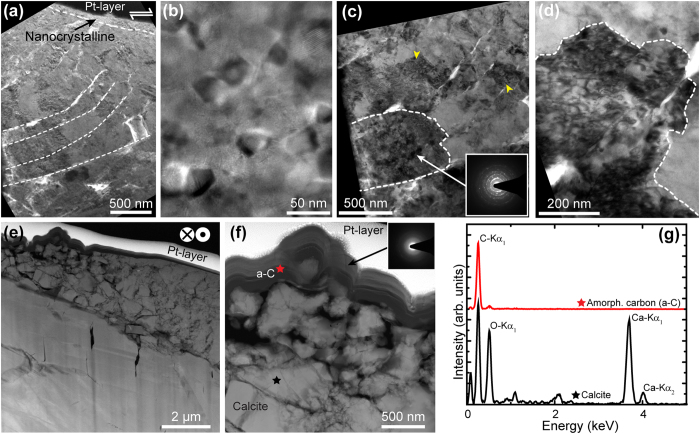
Rock volumes immediately below slip surfaces after (a to d) 1.5 mm and (e to f) 5 mm displacement. All images originate from FIB-SEM specimens cut perpendicular or parallel to the slip direction (shear sense arrows in Figs (**a**,**e))**. (**a**) Calcite grains cracked along bend cleavage planes (dashed lines). (**b**) High-resolution TEM image showing a nanograin (grain size: 5–50 nm) volume immediately below the slip surface, within the nanocrystalline domain. (**c**) Inter-cleavage crystal domains (dashed lines) exhibit complex TEM diffraction contrast due to a high degree of crystal defects, i.e. dislocations. High dislocations domains (arrow heads) remain either crystallographically coherent or develop a polycrystalline (see SAED pattern) mosaicism nanostructure, dashed area in c. (**d**) The high defect domains terminate abruptly into domains of low defect density. (**e**) After 5 mm displacement a semi-coherent amorphous carbon **a–c** layer develops across the slip surface. (**f**) Enlargement of Fig. (**e)** showing compositional layering of **a–c** and CaO-bearing phase immediately beneath the slip surface. Both Figs (**e**,**f)** are HAADF-STEM images. (**g**) EDS analysis confirms the presence of (**a–C**) in (**f**). Stars denote the analysed areas.

**Figure 5:**
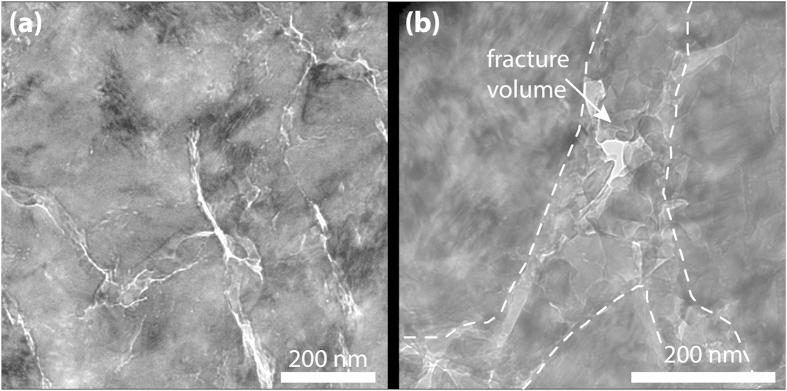
Microstructures after 1.5 mm slip. **(a)** Extensive micro-crack network associated to domains of high defect content (high diffraction contrast). (**b**) Numerous nanograins develop within the fracture volumes (bright-field TEM images).

In contrast with the pre-sheared rock surfaces, no exfoliation and surface cracking is observed on the sheared surface of the experiment stopped at *δ*_tot_ = 1.5 mm (s915-1, cf. Fig. 3a with b), where the deformation zone is 10–20 μm thick and shows a gradual increase in the degree of crystallinity away from the surface into the bulk rock material (Fig. 3e). We identified three domains between the sliding surface and the pristine calcite grains (dashed lines in Fig. 3e). Domain 1 consists exclusively of nanograins with a grain size of 5–100 nm (Fig. 4a,b; Supplementary Fig. S4 online). Domain 2 exhibits grains that are characterized by microfractures and closely spaced (~200 nm apart) cleavage bending planes (dashed lines in Fig. 4a). In domain 2 calcite areas in between cleavage planes exhibit intense mosaicism (Fig. 4c) alternating between areas that are nearly dislocation free to those that exhibit a high dislocation density (dashed region and domains indicated by yellow arrow heads in Fig. 4c and high diffraction contrast, HDC, in Fig. 4d). Although the HDC areas are crystallographic coherent with the surrounding crystal domains the ring-shaped nature of the selected area electron diffraction (SAED) patterns implies that these areas are polycrystalline, i.e. formed by individual nanograins (inset in Fig. 4c). In addition, nanograin formation is associated with intense micro- and nano-cracking (Fig. 5) and cleavage plane parting (dashed lines, Fig. 4a). This is in agreement with previous natural carbonate fault rock observations^26^. Domain 3 consists of subparallel cleavage planes spaced 2–3 μm apart from and merging into the pristine calcite grains (Fig. 3e) and is free of nanograins.

The sheared surface of the experiment stopped at δ_tot_ = 5 mm (s761, Fig. 3c) is characterised by a large number of smooth sliding surfaces (Supplementary Fig. S2 online) and presents features similar to those of the experiment stopped after δ_tot_ = 1.5 mm (c.f. Fig. 3b,c). However, flat slip micro-patches are covered by an amorphous carbon (a–C) nanofilm (Fig. 3f and HAADF-STEM image in Fig. 4e). Amorphous-C was identified by EDS and SAED analysis (red curve in Fig. 4g) on a selected area of the sheared zone (indicated with a red star in Fig. 4f). The EDS shows the disappearance of peaks corresponding to Ca (black curve in Fig. 4g), which are detected in a region about 500 nm below the sheared surface (black star in Fig. 4f). Calcite nanograins make up a discrete layer beneath the slip surface and are interspersed with and rimmed by a-C nanofilms (Fig. 4f).

## Discussion

Microstructures and reaction products summarized in Figs 3 and 4 show evidence for a strong structural disorder: (1) internal grain fragmentation and dramatic grain size reduction (mosaicism, cleavage bending) without major changes in calcite habit (Fig. 4a,c), as well as (2) CO_2_ emission and a-C formation (Figs 2 and 4e,f). CO_2_ emission triggered by a decarbonation reaction suggests the achievement of local temperatures (*T*) in excess of 650 °C ref. 37 after 1.5 mm of slip. In addition the a–C formation likely results from CO_2_ decomposition (CO_2_(g) → C(s) + O_2_(g)) under either highly compressed (several GPa), anoxic conditions^38^ or by thermochemical vapour deposition of C in the presence of H_2_ ref. 39. In our experiments the anoxic conditions are attained by separating the (back) reaction products if O_2_ escapes faster than the time needed for oxidation to occur^40^, suggesting that the mechanism leading to a–C deposition is short-lived (<ms). Alternatively, the reaction leading to carbon deposition is either CH_4_(g) = C(s) + 2H_2_(g), where CH_4_ forms by the hydrogenation of calcite (CaCO_3_(s) + 4 H_2_(g) → CaO(s) + CH_4_(g) + 2H_2_O (g)), or from the reduction of CO_2_ by CO_2_(g) + 2H_2_(g) → C(s) + 2H_2_O(g) at T > 580 °C. H_2_ in both cases results from the electrolysis of the acidified surface-adsorbed H_2_O (room-humidity). Given that the slow kinetics of the electrolyses, the short duration of the process can occur, in the absence of a catalysts, only under an enhanced chemical reactivity. The detection of molecular fragments of hydrocarbons (C_x_H_y_, Fig. 2) seems to support a mechanism of thermochemical vapour deposition. Hydrocarbons might have formed also from the reaction of CO and H_2_ where CO-rich gases proceeds through the intermediate step CO_2_ → CO + 1/2O_2_. In this case carbon forms spontaneously by the disproportionation of CO according to 2CO(s) → C(s) + CO_2_(g). This reaction is highly exothermic and characterized by high activation energy. As a consequence the a–C formation can occur only on chemically active new surfaces by non-equilibrium process like CO adsorption^41^. Since we can only investigate post-deformation microstructures and measure gas emission relatively far from the shear zone the mechanism of a–C formation remains puzzling. However, all observations are indicative of an abrupt and local temperature rise (the CO_2_ emission and the presence of a–C material are consistent with T > 600 °C) possibly concomitant with stress transients of few GPa and a high chemical reactivity.

**Figure 6:**
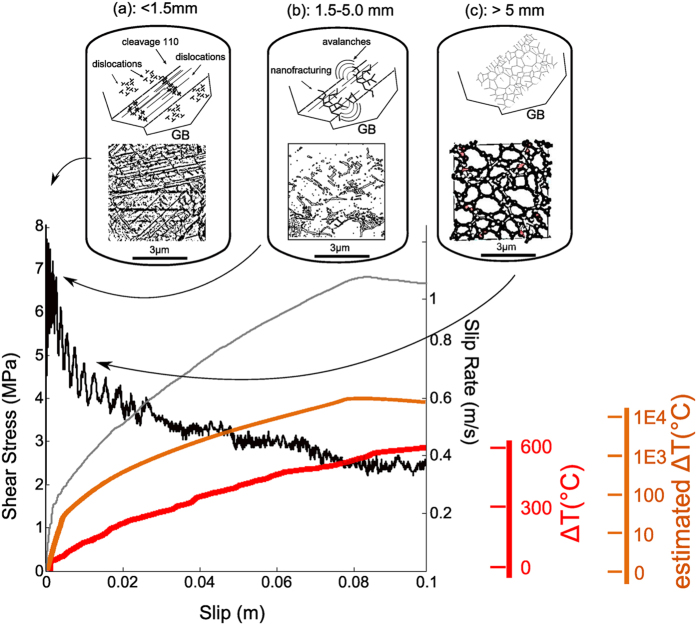
Evolution of microstructures (top diagrams), shear stress (gray in colour curve), slip-rate (dark gray curve), bulk temperature (red curve), estimated temperature from fast moving dislocation theory (Eq. 2, orange curve) in calcite marble with slip. **(a)** For slip <1.5 mm, new dislocations are emitted and pile up at the intersections with microstructural obstacles (e.g., cleavage planes, grain boundary GB). **(b)** For 1.5 mm < slip < 5 mm, dislocations are released in “avalanches” forming nano-grains, (**a–C)** and inducing CO_2_ emission; **(c)** for slip >5 mm, the presence of (**a–C)** at the asperity contacts may lubricate the fault. The estimated bulk temperature increase is <100 °C after 1.5 mm of slip but fast dislocation avalanches may result in a temperature increase of > 1000 °C.

An upper estimate of the bulk temperature increase in the slipping zone, due to the frictional heat exchanged along a sliding interface, can be obtained by^42^:


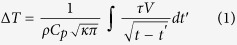


where κ is the calcite thermal diffusivity (2.4 10^−6^m^2^s^−1^), *ρ* is the calcite density (2711 kg m^−3^), *C*_*p*_ the calcite heat capacity (852 J kg^−1^ K^−1^). Conversely to the high temperature rise predicted from the microstructural and geochemical observations, the solution of Eq. (1) results in a bulk temperature increase of ~100 °C after 5 mm of slip (Fig. 6). This estimate is consistent with measurements obtained with an infrared thermo-camera in experiments performed on limestone under similar deformation conditions^21^. It has been proposed that grain size reduction to the nanoscale reduces the melting (and decarbonation) point temperature in ceramic materials^43^. The ratio (*K*) between the grain size and the atomic bond length controls the depression of the melting point: for *K* > 20, the melting point depression is negligible. The nanograins (Fig. 4b) produced in our experiments are ~10 nm in radius and considering a bond length in calcite to be ~0.13 nm yields a *K*-value of ~77. Thus, the expected melting (i.e., decarbonation for calcite) temperature depression in our experiments is of a few tens of °C at most. Moreover, nanograins may resulted from abrasion and wearing but the mechanics of abrasion and wearing is the same as the mechanics of grain fracturing^44^. Fracture theory predicts a limit to grain size reduction down to 1 μm in compression^45^. Smaller grain size can be achieved by sub-critical crack growth (low strain rates) and shock loading (high strain rates)^46^. However the slowness of the process of sub-critical crack growth (1 s to split a 1μm in size grain under a typical crack velocity of 10^−6 ^ms^−1^ in wet calcite^47^) compared to the short duration of the experiments (≪1 s) suggests that nanograins formed at conditions approaching those of a shock loading. This consideration is also in agreement with our observation that failure have initiated at many closely spaced points within a small volume where multiple fracture have grown simultaneously (Fig. 5). Energetic considerations on rock fragmentation mechanics predicts that the short and closely spaced fractures should have occurred in a cohesive rock under an instantaneous and local stress of the order of GPa and strain rates in excess of 10^5^ s^−1^ ref. 28.

A way to reconcile the above mineralogical and geochemical considerations (e.g. CO_2_ emission, a–C formation) with (1) nanograin formation, (2) abrupt temperature rise, (3) shear stress reduction at slip initiation and (4) the presence of a threshold in slip velocity to trigger frictional weakening, is to consider the microstructural processes operating at the initiation of slip. These microstructural processes have to provide the energy necessary for both frictional heating causing phase transformations (e.g. a–C) and brittle nanofracturing. Our microstructural, mineralogical and geochemical observations are all indicative of a high structural disorder that has occurred during a short time period within an extremely narrow zone. In particular, the internal fragmentation of calcite by fracturing (Fig. 5) and the high dislocation density as expressed by the mosaics structures (Fig. 4c,d) indicate that slip initiation is controlled by defect-related mechanisms, i.e. dislocation mobility. Indeed, the strength of the material and its plastic flow depends on how easily dislocations are created and destroyed and what energy threshold is required to move them. At high strain rates, stress relaxation occurring by the collective motion of discrete newly formed or pre-existent dislocations may be inhibited, changing the ductile failure behaviour typical of low strain rates into a brittle failure under highly dynamic conditions^33^. Moreover, at high strain rates, given the low thermal diffusivity of calcite, the kinetic energy of fast moving dislocations within a narrow shear band is (adiabatically) dissipated into heat. The exchange of kinetic energy into heat is thus accompanied by (1) an abrupt temperature increase, (2) the generation of new defects, (3) chemical disorder in the crystal structure and (4) brittle fracturing. Although it is challenging to monitor dislocation motion *in-situ* during deformation at high strain rates, the predictions of the fast-moving dislocations theory^48^,^49^ are in agreement with our microstructural and geochemical observations (e.g. nanofracturing, thermal decomposition and amorphization). Moreover, the fast moving dislocation theory necessarily introduces a critical strain rate at which the mechanism of thermal softening is hindered leading to brittle failure. The critical strain rate, as discussed later, may be related to the direct experimental observation of a critical velocity for the initiation of fault weakening.

The microstructural observations summarized by Figs 3 and 4 and the reaction products are analogous to those attributed to shock-induced metamorphism in calcite^50^ suggesting that similar processes may occur within a seismically active shear zone. Numerical models proposed for shock loading and meteoritic impacts^32^,^51^ are based on the theory of fast-moving dislocations and a similar approach was theorized in the framework of high speed friction experiments^32^.

The mechanics of fast moving dislocations and the way dislocations trigger flash heating and weakening in carbonate-bearing faults, can be summarized in three stages (Fig. 6).

### Birth of dislocations

The sharp change in strain-rate due to an increase in velocity results in a sudden change in the state of stress at grain boundaries or pre-existing defects and the nucleation of new dislocations. Most of the strain is accommodated by slip along cleavage planes and newly-formed dislocations migrating through the crystal structure until reaching microstructural obstacles (e.g., cleavage planes, micro-cracks, twins and grain boundaries), producing intragranular micro-fractures along crystallographically controlled weaknesses^52^,^53^.

### Pile up of dislocations

Dislocations emitted from the same source will pile up at the microstructural obstacle, increasing the dislocation density. The stress at the head of the pile up will subsequently increase until it reaches the yielding point (*σ*_*y*_) given by the Hall-Petch relationship (*σ*_*y*_ = *σ*_*i*_ + *k*_*HP*_*d*^*−1/2*^), where *σ*_*i*_ is the flow stress related to the density of dislocations accumulated in dislocation boundaries, *d* is the grain size and *k*_*HP*_ is a constant derived empirically for each material which is also known as the Hall - Petch parameter^54^.

### Dislocation avalanches

Piled up dislocations are instantaneously unpinned at the critical stress of the strongest microstructural obstacle, resulting in catastrophic dislocation avalanches^55^. The kinetic energy of fast-moving dislocations is converted into heat through the viscous damping by electrons and phonons in the crystal structure^48^,^49^. The avalanches will result in an ultrafast conversion of the kinetic energy from dislocations into heat and will induce the formation of “dislocation hot-spots” (i.e., the emission of newly formed, locally sourced dislocations) and fast stress release^56^. The temperature increase (Δ*T)* inside the crystal, when the piled up dislocations are abruptly released, can be estimated by^55^:


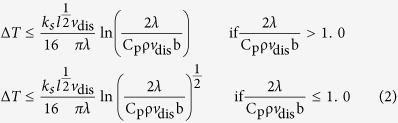


where 
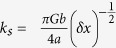
 is the Hall-Petch microstructural shear stress intensity for the pile up, *λ* the thermal conductivity, *l* the average grain size of calcite, *a* = 2(1 − *v*)(2 − *v*) with *v* being the Poisson’s ratio, *v*_*dis*_ the average velocity of dislocations inside the grain, *G* the shear modulus, *b* the Burgers vector and 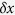 the dislocation spacing at the tip of the pile up. If the micro-cracks are formed by dislocation pile ups at grain boundaries, the number of micro-cracks should be proportional to the dislocation density *ρ*_*dis*_ ref. 57 which, following the Orowan’s relation, leads to a strain rate of:


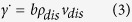


where *ρ*_*dis*_corresponds to a fraction *p,* ranging between [0–1], of the entire population of dislocations, which is moving at *v*_*dis*_. Given the slipping zone thickness *h* = [0.5–10] μm (domains 1 and 2 in Fig. 3e,f) and V* ~0.1 ms^−1^ (Table 1) the average strain rate across the slipping zone when large frictional weakening occurs is 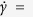
*V/h *~ 10^4^ s^−1^. For Carrara marble and shocked calcite (*ρ*_*dis*_ = [10^12^–10^14^ ] m^−2^ ref. 58, *l* = 0.3 mm, *λ* = 5.54 (Ns^−1^K^−1^), *a* = 2.38, *G* = 35 *G*Pa ref. 59, *b* = 0.49 nm ref. 58 and 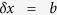 at cracking^55^) the temperature increase calculated from Eq. (2) for a calcite crystal after 1.5 mm of slip is up to 10^4^ K, i.e. orders of magnitude higher than the bulk temperature increase estimated in the slipping zone from Eq. (1) (Fig. 6).

Similarly, it is possible to estimate the critical strain rate above which thermal instability of a crystal occurs. The critical strain rate is related to dislocation mobility which is in turn affected by the resistance to dislocations motion including phonon scattering, impurity drag and lattice periodicity (e.g. the Peierls potential). The resistances can be lowered by an amount of energy Δ*G* given by thermal fluctuations; thus the rate at which the dislocations pass the energetic barrier (aided by the energy contribution Δ*G*) increases with temperature and the dislocation mobility will be enhanced. However, as the strain rate increases, there is less time available for the dislocations to overcome the energetic barrier until, above a critical strain rate 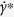, thermal activation is rendered ineffective^48^,^49^:


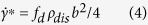


where *f*_*d*_ is the vibrational frequency of the dislocations (~10^11^s^−1^) ref. 60. Using Eq. (4), 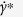
~ 10^3^–10^5^ s^−1^ in the case of calcite and, given a homogeneously distributed strain in a slipping zone of thickness *h* = [0.5–10] μm, V* is approximatively in a range of [0.05–1] ms^−1^. The lower estimate of V* is compatible with the V* measured in our experiments (~0.10 ms^−1^).

Although the conceptual framework of fast-moving dislocations for calcite is still incomplete, the existence of a critical strain rate may also justify the initial sliding at high shear stress where thermal softening, occurring by thermal activation, relaxes the stress singularity at the crack tip by viscous flow^61^. However, for 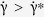 the mechanism of stress relaxation is inhibited and the ductile behaviour of calcite, typical at low strain rates near the crack tip, turns into a brittle behavior^33^. As a consequence, calcite is more prone to release stress by fracturing at high sliding velocities. The fast stress release separates highly compressed from uncompressed domains within the calcite grains and induces grain fragmentation and mosaicism (Figs 4a–d and 5). The ultimate fragment size is determined by the minimum grain size that can sustain dislocation pile up^62^, corresponding to a few nanometers in our experimental conditions. The high rate of dislocations production results in grain size reduction and in an increase in specific surface area enhancing chemical reactivity^63^. The latter might allow the thermochemical vapour deposition of amorphous carbon in the presence of H_2_ and the observed shorter slip weakening distance in the presence of liquid H_2_O (Fig. 4 in Violay *et al.*^9^). The brittle failure induced by the fast moving dislocations could also explain nanograin formation in both natural^25^,^26^,^27^,^28^ and experimental fault zones^16^,^18^,^19^,^20^,^21^,^23^,^24^,^25^. Nanograins formed under mechanical treatment are composed of nano-crystals separated by interfaces or amorphous layers^63^. Once slip ceases, the temperature rapidly decreases and the chemical reactivity is strongly reduced. Surface reconstruction and chemical reactions with the environment result in the layered microstructures of the slip surface (Fig. 4f).

Once generated, fast-moving dislocations act as a viscous component of the rheology of calcite grains with two major consequences for the shear stress dependence with strain rate. Firstly, the theory of fast-moving dislocations predicts a strain rate threshold V* to trigger a dislocation avalanche, which results from the competition between thermal relaxation and the thermal excitation of dislocation motion. V* in the present experiments was ~0.10 m s^−1^, which is compatible with previous studies^8^,^9^,^21^ and estimates from Eq. (4). The high strain rates associated with seismic deformation conditions might be responsible for the thermal activation of flash heating. Secondly as thermal excitation increases with temperature, a positive feedback is established between the temperature rise due to dislocation kinetics damping and the viscous dissipation itself which sustains fast dislocation motion during slip initiation.

The transition from slip initiation to frictional strength reduction and fault weakening (δ > 5 mm, Fig. 6c) is triggered by fast moving dislocations and may be due to either a grain-size dependent process (e.g. diffusion creep, grain boundary sliding, superplasticity^22^,^23^,^24^) or to the presence of discontinuous fault micro-patches covered by a nano-layer of a–C.

Amorphous-C has been previously indicated as a lubricant for fault weakening^64^,^65^ due to its low friction coefficient value (~0.15) ref. 66. This lubrication mechanism developed at the asperity contacts would be equivalent in carbonate-bearing rocks to the formation of asperity scale melt drop patches responsible for abrupt initial weakening in silicate-bearing rocks (flash heating and melting^2^,^3^,^4^,^5^,^6^,^7^,^8^,^9^). Given that amorphization is a low temperature type of melting^67^, the presence of an amorphous phase results in a strong inverse non-linear dependency of viscosity (and, as a consequence, on shear stress) on temperature. This dependence is able to explain the shear stress recovery upon sample deceleration (*τ*_*f*_ in Table 1).

Recent studies have proposed that grain boundary sliding in nano-grain built slipping zones results in frictional strength reduction for δ > 0.5 m in carbonate-bearing faults^23^,^24^. Nanograin formation has been attributed to phase transformations accompanied by CO_2_ release^19^ which is in agreement with the observations reported here. Although the discussion on processes activated at large fault slip is beyond the scope of this paper, grain boundary sliding may operate in conjunction with a–C at slip initiation and become the dominant weakening mechanism with progressive slip. According to the above scenario, fast-moving dislocations are the microphysical process responsible for the abrupt temperature rise at the asperity scale which leads to nanograins and a-C formation in carbonate-bearing rocks and possibly to flash melting in silicate-bearing rocks. In this interpretation, fast-moving dislocations are a general microphysical mechanism for flash heating at the initiation of seismic slip.

## Additional Information

**How to cite this article**: Spagnuolo, E. *et al.* Fast-moving dislocations trigger flash weakening in carbonate-bearing faults during earthquakes. *Sci. Rep.* **5**, 16112; doi: 10.1038/srep16112 (2015).

## Supplementary Material

Supplementary Information

## Figures and Tables

**Table 1:** Summary of experimental results.

	τ_p_ (MPa)	τ_min_ (MPa)	τ_f_ (MPa)	V_targ_ (ms^−1^)	V_max_ (ms^−1^)	V* (ms^−1^)	δ (mm)
s257	7.46	1.02	2.80	1.00	1.00	0.09	2000
s301	6.17	0.51	3.17	6.50	6.50	0.10	20000
s763	6.62	2.56	3.67	1.00	0.79	0.13	50
s765	7.70	1.23	2.79	1.00	1.00	0.17	500
s767	7.17	5.60	5.70	1.00	0.24	0.06	5
s769	6.30	1.14	3.21	1.00	1.00	0.13	5000
s761	8.40	5.70	5.90	1.00	0.32	0.05	5
s915-1	8.25	6.39	7.44	1.00	0.20	0.06	1.5
s1187	7.90	5.65	7.50	1.00	0.18	0.14	1.5
	9.00	5.70	7.50	1.00	0.18	0.14	1,5
	9.20	5.90	8.60	1.00	0.20	0.13	1.5
	8.36	4.19	5.224	1.00	0.68	0.21	50
s915-0							0
*mean*	7.71					0.12	
*sigma*	1.00					0.05	

Acronyms and symbols: *τ*_*p*_: peak of frictional strength; *τ*_*min*_: minimum frictional strength; *τ*_*f*_: peak of frictional strength upon deceleration; *V*_*targ*_: target velocity; V*: critical velocity for weakening at slip initiation; *δ*: total slip. *σ*_*n*_ = 10 MPa in all the experiments. See text for explanation.
