# Correction: Fast-moving dislocations trigger flash weakening in carbonate-bearing faults during earthquakes

**DOI:** 10.1038/s41598-026-35668-2

**Published:** 2026-02-11

**Authors:** Elena Spagnuolo, Oliver Plümper, Marie Violay, Andrea Cavallo, Giulio Di Toro

**Affiliations:** 1 Sezione di Sismologia e Tettonofisica, Istituto Nazionale di Geofisica e Vulcanologia, Via di Vigna Murata 605, Roma, Italy; 2 Department of Earth Sciences, Utrecht University, Budapestlaan, 4, P.O. Box 80.021, 3584 CD Utrecht, the Netherlands; 3 EPFL, LEMR, Station 18, 1015 Lausanne, Switzerland; 4 Dipartimento di Geoscienze, Università di Padova, Via G. Gradenigo 6, 35131 Padova, Italy; 5 School of Earth, Atmospheric and Environmental Sciences, Manchester University, Oxford Street, M13 9PL Manchester, United Kingdom

Correction to: *Scientific Reports* 10.1038/srep16112, published online 10 November 2015

This Article contains an error in equation 2, which was incorrectly typeset as:2

The equation 2 has now been corrected and reads:

$$\Delta T \le \frac{{k}_{s}{l}^\frac{1}{2}\:{v}_{dis}}{16\:\pi\: \lambda } \times \left\{\begin{array}{c}\mathrm{ln}\left(\frac{2\lambda }{{C}_{p}\rho\:{v}_{dis}\:b}\right)\quad if (\frac{2\lambda }{{C}_{p}\rho\: {v}_{dis}\:b} > 1.0)\\ {\left(\frac{2\lambda }{{C}_{p}\rho\:{v}_{dis}\:b}\right)}^{1/2} \quad if (\frac{2\lambda }{{C}_{p}\rho\:{v}_{dis}\:b} \le 1.0)\end{array}\right.$$

