# Supplementary material for: Fast-moving dislocations trigger flash weakening in carbonate-bearing faults during earthquakes
Source: Sci Rep. 2015 Nov 10;5:16112. doi: 10.1038/srep16112 (PMC4639853; doi:10.1038/srep16112)
Supplement: Supplementary Information [file srep16112-s1.pdf]

Supplementary figures and captions for:

Fast-moving dislocations trigger flash weakening in carbonate-bearing faults during earthquakes

Elena Spagnuolo*, Oliver Plümper, Marie Violay, Andrea Cavallo, Giulio Di Toro

*To whom correspondence should be addressed. Email: elena.spagnuolo@ingv.it

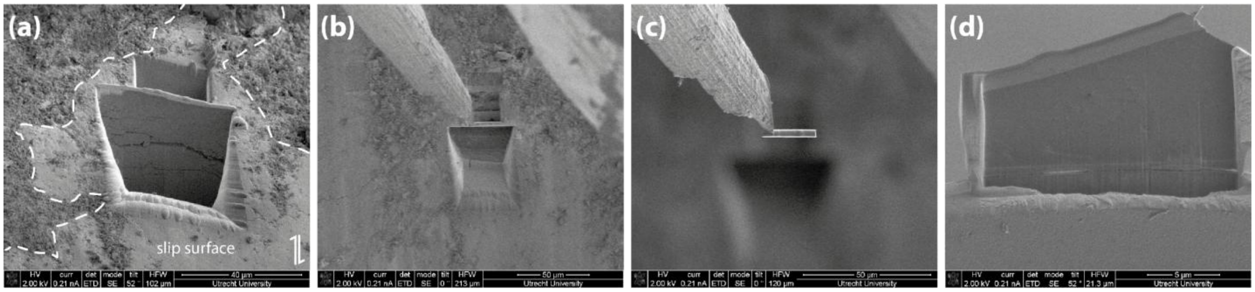

Supplementary Figure S1: (**a**) FIB-SEM cross-section of a rock surface after 1.5 mm slip ready for high-resolution SEM investigation. After completion of the SEM investigation a second pit is milled that allows to lift out the cross-section from the bulk rock sample (see **b** and **c**). (**d**) FIB section for TEM investigation mounted onto a Cu-grid.

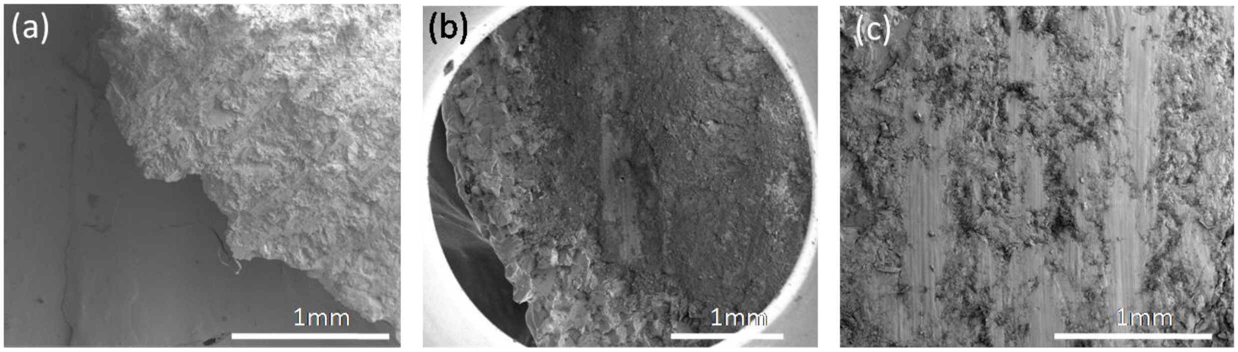

Supplementary Figure S2: (a) The pre-sheared surface of pre-sheared experiment (s915-0) is decorated by an incoherent distribution of flattened patches due to sample preparation (surface grinding and polishing with diamond bits). **(b)** After 1.5 mm of slip (s915-1) the surface presents a large number of smooth, continuous flat patches, extending for several millimetres across the surface. **(c)** After 5 mm of slip (s761) the flat patches are larger, smoother and more continuous with respect to (b).

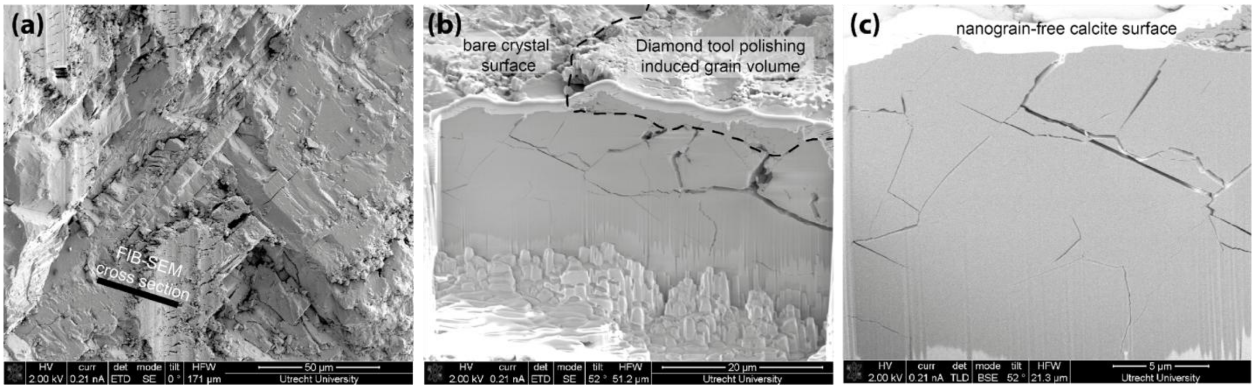

Supplementary Figure S3:(**a**) Secondary electron images of a pre-sheared rock surface (s915-0) and location of the corresponding FIB-SEM cross-section. All pre-sheared rock surfaces are dominated by the exposure of nanograin-free calcite grain surfaces (**b** and **c**). Sporadically, pre-experiment diamond tool polishing generated minor amounts of flat, discontinuous, polycrystalline patches. they did not contain amorphous carbon.

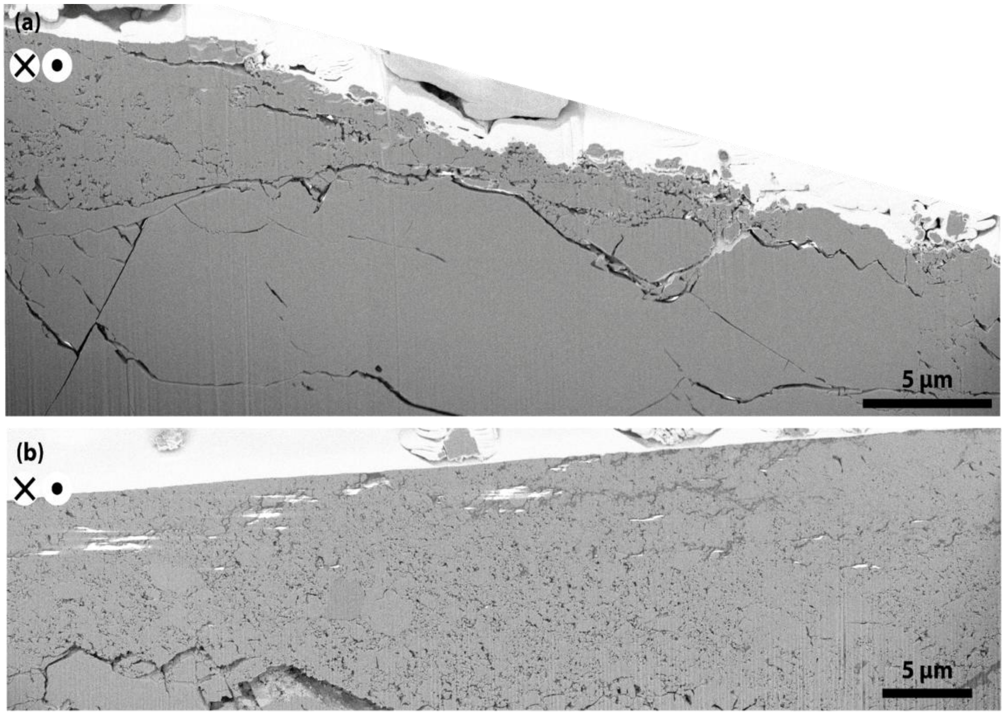

Supplementary Figure S4: (**a**) and (**b**) are cross-sectional views cut perpendicular to slip direction of sample collected after 1.5 mm slip (s915-1). The slip zones are made up of large micro- to nanograin volumes covering large areas of the rock surface. Calcite grains beneath the experimentally-produced fault gouge volume develop both cleavage- and grain boundary-controlled cracks as well as cracks across grains independent of crystallographic orientations.
